# Remifentanil-induced alterations in neutrophil numbers after surgery

**DOI:** 10.1186/s40981-016-0031-z

**Published:** 2016-04-02

**Authors:** Toshiichro Inagi, Hideko Hoshina, Manzo Suzuki, Miki Wada, Hiroyasu Bito, Atsuhiro Sakamoto

**Affiliations:** 1 Department of Anesthesiology, Musashikosugi Hospital Nippon Medical School, 1-396 Kosugi-cho Nakahara-ku, Kawaski, Kanagawa 211-8533 Japan; 2 Department of Breast Surgery, Musashikosugi Hospital Nippon Medical School, Kanagawa, Japan; 3 Department of Aneasthesiology, Nippon Medical School, 1-1-5 Sendagi Bunkyo-ku, Tokyo, 113-8102 Japan

**Keywords:** Neutrophil counts, Remifentanil, Surgery, Anesthesia

## Abstract

**Background:**

Neutrophils are the first line of defense against invasive microorganisms during and after surgery. There is a possibility that different opioid analgesics used during surgery have different effects on the leucocyte count. We retrospectively analyzed the numbers of leucocytes, neutrophils, and lymphocytes just after surgery in patients who received remifentanil-based anesthesia and those who received fentanyl-based anesthesia.

In female patients who underwent modified mastectomy or simple mastectomy with resection of a lymph node(s) or with biopsy of a sentinel lymph node(s) between January 2010 and December 2013 (*n* = 83), propensity score pairwise matching was performed according to the patient’s age and procedure, and forty patients (Remifentanil group and Fentanyl group; *n* = 20 each) were analyzed.

**Findings:**

Postoperative numbers of leucocytes and neutrophils were significantly lower in patients who received remifentanil-based anesthesia than in those who received fentanyl-based anesthesia (*p* = 0.03, *p* = 0.014; leucocytes and neutrophils, respectively). The increases in the numbers of leucocytes and neutrophils were significantly lower in the patients in the remifentanil group (*p* = 0.009, *p* = 0.0046; increase in leucocytes and neutrophils, respectively).

**Conclusions:**

In conclusion, remifentanil-based anesthesia attenuates postoperative leucocyte and neutrophil counts. It is unknown whether this phenomenon indicates the possibility of immunosuppression. Further studies are required.

## Findings

### Introduction

Neutrophils are the first line of defense against invasive microorganisms during and after surgery. The numbers of leucocytes, neutrophils and lymphocytes may change in response to surgery due to various hormones, cytokines and acute phase reactants [1].

Neutrophils are produced within hematopoietic cords interspersed within venous sinuses of the bone marrow, and the number of circulating neutrophils is maintained by a fine balance among granulopoiesis, bone marrow storage and release, intravascular margination, clearance, apoptosis and destruction [2]. The principal regulator of granulopoiesis is granulocyte colony stimulating factor (G-CSF) [3]. Interleukin (IL)-6 and IL-8 are also known to promote neutrophil production in vitro and in vivo [4, 5].

The production of immune mediators is associated with the extent of surgical trauma, and is also influenced by the anesthesia technique [6, 7]. Remifentanil is a newly developed analgesic that possesses extremely rapid clearance. An in vitro study suggested that remifentanil decreased expression of inflammatory cytokines such as TNF-α, IL-6 and IL-8 induced by lipopolysaccharide (LPS) in human neutrophils [8].

These results suggest that the different opioid analgesics used during surgery may have different effects on the leucocyte count. We retrospectively analyzed the numbers of leucocytes, neutrophils, and lymphocytes just after surgery in patients who received remifentanil-based anesthesia and those who received fentanyl-based anesthesia.

## Methods

The records of female patients who underwent modified mastectomy or simple mastectomy with resection of a lymph node(s) or with biopsy of a sentinel lymph node(s) between January 2010 and December 2013 were retrospectively investigated. This study was approved by the ethics committee of Musashikosugi Hospital Nippon Medical School. Exclusion criteria included patients who received chemotherapy before surgery, patients who presented with a febrile symptom before the surgery (C-related protein >0.3), and patients over the age of 75 years. Patients whose surgery lasted less than 80 min or longer than 181 min were excluded.

The following data were collected and compared between the patients who received remifentanil-based anesthesia and those who received fentanyl-based anesthesia: patients’ age, height, weight, duration of surgery, amount of infusion, blood loss, and total amount of opioid analgesic (fentanyl, remifentanil, or both) administered during surgery. The systolic blood pressure before the surgery, highest systoli**c** blood pressure during surgery, systolic blood pressure just after the surgery, and heart rates at the respective systolic blood pressure were recorded. The mean infusion rate of remifentanil (μg/kg/h) was calculated by dividing the total amount of remifentanil administered by the patient’s weight and the duration of the surgery. Preoperative and postoperative counts of leucocytes, neutrophils and lymphocytes were recorded. The increases in numbers of leucocytes, neutrophils, and lymphocytes were calculated by subtracting the preoperative value from the respective postoperative value. Patients who received additional pain medicine (e.g., NSAIDs) were noted.

### Statistical analysis

To reduce the selection bias on the number of immune cells, we performed a propensity score pairwise matching of patients maintained with remifentanil with patients maintained with fentanyl, using patient’s age and procedure. As the propensity score-matched analysis was selected to reduce preoperative confounding factors, the propensity score was constructed with the aid of a logistic regression model using preoperative variables. Patients maintained with remifentanil whose propensity scores deviated by >0.02 from those of patients maintained with fentanyl, were considered unmatched.

Results are shown as mean ± standard deviation. The significance of differences in categorical values and numerical values were analyzed using chi-squared test and unpaired *t*-test, respectively. The correlation between numerical parameters was analyzed using linear regression analysis. Significance was set at *p* < 0.05.

## Results

**Fig. 1:**
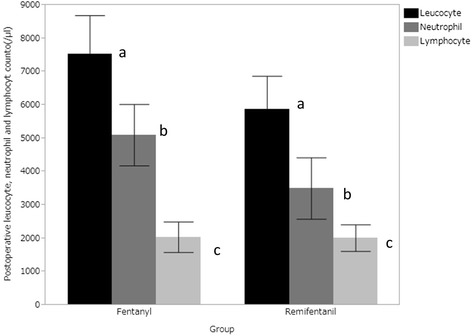
Comparison of the postoperative numbers of leucocytes (black bar), neutrophils (gray bar) and lymphocytes (white bar) between the patients who received fentanyl-based anesthesia (*n* = 20) and those who received remifentanil-based anesthesia (*n* = 20). The leucocyte count (a: *p* = 0.03) and neutrophil count (b: *p* = 0.014) were significantly lower in patients who received remifentanil-based anesthesia than in those who received fentanyl-based anesthesia. Error bars represent the standard deviation. There was no significant difference in the number of lymphocytes between the two groups (c: *p* = 0.92)

**Fig. 2:**
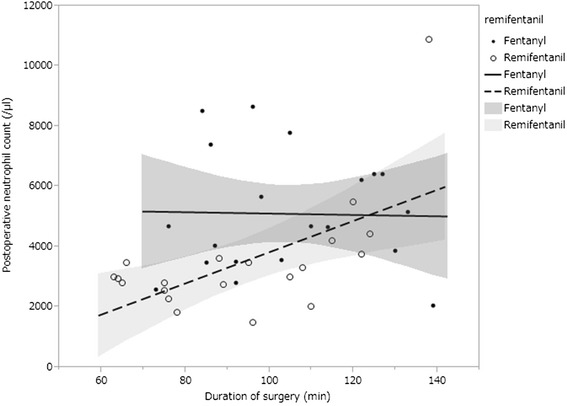
Correlations between the postoperative neutrophil count and duration of surgery in fentanyl group (thick line and dark shadow), and between the postoperative neutrophil count and duration of surgery in remifentanil group (dotted line and light shadow). The dark shadow represents the 95 % confidence interval (CI) in fentanyl group and the light shadow represents the 95 % CI in remifentanil group. Neutrophil count =5335-2.2*duration of surgery (min). *R* ^2^ = 0.00, *P* = 0.92 (Fentanyl group). Neutrophil count = -1349 + 51.8*duration of surgery (min). *R* ^2^ = 0.36, *P* = 0.0044 (Remifentanil group)

Eighty-three patients underwent modified mastectomy or simple mastectomy with resection of a lymph node or with biopsy of a sentinel lymph node met the study criteria. Of these, 36 patients received fentanyl-based anesthesia and 47 patients received remifentanil-based anesthesia. All patients received propofol and rocuronium, and trachea was intubated. Anesthesia was maintained by sevoflurane, propofol had not been used for maintenance. Blood samples had been obtained within 2 h after emergence from anesthesia in all patients. The characteristics of the patients who received remifentanil-based anesthesia or fentanyl-based anesthesia are summarized in Table 1. After propensity matching, we obtained 40 patients for analysis, which included 20 patients in the remifentanil group who received remifentanil-based anesthesia, and 20 patients in the fentanyl group who received fentanyl-based anesthesia. No patients in neither fentanyl nor remifentanil group developed problem in wound healing process such as surgical site infection. No patients received catecholamine as inotropes, only ephedrine had been used. As shown in Table 2, there is no difference in number of patients who received ephedrine and in amount of ephedrine. Postoperative numbers of leucocytes and neutrophils were significantly lower in remifentanil group than fentanyl group (leucocytes, 7521 ± 2492 vs. 5873± 2123, *p* = 0.03; neutrophils, 5098± 1964 vs. 3499 ± 1970, *p* = 0.014) (Fig. 1). There was no significant difference in the number of lymphocytes between the two groups (Fig. 1). Among the 20 patients in the remifentanil group, 13 patients received additional fentanyl. In the remifentanil group, there were no differences in the numbers of leucocytes, neutrophils and lymphocytes between the patients who received additional fentanyl and those who did not. The postoperative number of of neutrophils was correlated with the duration of surgery only in the remifentanil group (R2 = 0.36, *p* = 0.004) (Fig. 2).

**Table 1:** Characteristics of the patients who received remifentanil-based anesthesia or fentanyl-based anesthesia

		Remifentanil (*n* = 47)	Fentanyl (*n* = 36)	*P* value
Age (y)		57 ± 11	51 ± 10	0.025
Height (cm)^a^		156 ± 6	154 ± 7	0.21
Weight (kg)^a^		55 ± 8	55 ± 10	0.98
Mastectomy: partial mastectomy		26:21	13:23	0.39
SLNB or RLN^b^		23:24	21:15	0.08
Received flubiprofen		19	15	0.9
Total amount of remifentanil (μg/kg)^a^		23.5 ± 11.4	0 ± 0	<0.0001
Total amount of fentanyl (μg/kg)^a^		2.2 ± 2.8	5.5 ± 2.5	<0.0001
Duration of surgery (min)^a^		111 ± 33	122 ± 30	0.1
Mac-hour(h)		2.1 ± 0.5	2.6 ± 0.7	0.01
Total amount of ephedrine (mg)		3 ± 3.8	1.3 ± 2.2	0.06
Number of Patients received ephedrine (n)		21	12	0.36
Blood pressure (mmHg)	Preope	144 ± 24	139 ± 25	0.36
	Highest	122 ± 17	131 ± 18	0.019
	Lowest	82 ± 7	88 ± 10	0.0024
	Postope	133 ± 17	133 ± 24	0.95
Heart rate (b/min)	Preope	73 ± 13	70 ± 13	0.35
	At highest BP	61 ± 9	64 ± 10	0.14
	At lowest BP	55 ± 7	56 ± 7	0.42
	Postope	74 ± 12	72 ± 14	0.43
Total amount of infusion (ml)^a^		1250 ± 385	1325 ± 390	0.4
Preoperative leucocyte count (/μl)^a^		5726 ± 1656	5536 ± 1405	0.63
Preoperative neutrophil count (/μl)^a^		3270 ± 1230	3210 ± 1215	0.83
Preoperative lymphocyte count (/μl)^a^		1910 ± 690	1820 ± 545	0.51
Preoperative monocyte count (/μl)^a^		260 ± 80	270 ± 110	0.64

^a^Mean ± SD

^b^ *SLNB* sentinel lymph node biopsy, *RLN* resection of lymph node

**Table 2:** Characteristics of the patients who received remifentanil-based anesthesia or fentanyl-based anesthesia after propensity matching

		Remifentanil group (*n* = 20)	Fentanyl group (*n* = 20)	*P* value
Age (y)		54 ± 11	52 ± 12	0.68
Height (cm)^a^		157 ± 7.5	152 ± 5	0.38
Weight (kg)^a^		54 ± 9	56 ± 11	0.58
Mastectomy: partial mastectomy		5:15	6:14	0.72
SLNB or RLN^b^		19:1	18:2	0.54
Received flubiprofen		19	15	0.9
Total amount of remifentanil (μg/kg)^a^		20.1 ± 9.4	0 ± 0	<0.0001
Total amount of fentanyl (μg/kg)^a^		1.9 ± 1.9	5.4 ± 2.1	<0.0001
Duration of surgery (min)^a^		94 ± 23	103 ± 20	0.14
MAC-hour(h)		2.2 ± 0.5	2.7 ± 0.7	0.01
Total amount of ephedrine (mg)		3 ± 3.5	1.2 ± 2.1	0.06
Number of Patients received ephedrine (n)		9	5	0.32
Blood pressure (mmHg)	Preope	137 ± 20	139 ± 24	0.8
	Highest	116 ± 15	135 ± 17	0.0016
	Lowest	80 ± 7	87 ± 10	0.02
	Postope	130 ± 16	134 ± 25	0.64
Heart rate (b/min)	Preope	76 ± 16	69 ± 12	0.10
	At highest BP	63 ± 10	65 ± 11	0.22
	At lowest BP	55 ± 7.5	56 ± 7.5	0.8
	Postope	74 ± 15	72 ± 10	0.48
Total amount of infusion (ml)^a^		1235 ± 290	1360 ± 460	0.3
Preoperative leucocyte count (/μl)^a^		5460 ± 1460	5622 ± 1587	0.74
Preoperative neutrophil count (/μl)^a^		3169 ± 1192	3274 ± 1264	0.78
Preoperative lymphocyte count (/μl)^a^		1845 ± 663	1857 ± 619	0.95
Preoperative monocyte count (/μl)^a^		265 ± 81	267 ± 106	0.94

^a^Mean ± SD

^b^ *SLNB* sentinel lymph node biopsy, *RLN* resection of lymph node

## Discussion

In the present study, we observed that postoperative counts of leucocytes and neutrophils were significantly lower in the patients who received remifentanil-based anesthesia than in those who received fentanyl-based anesthesia. Because the number of lymphocytes and increase in the number of lymphocytes from the preoperative value did not differ between those who received remifentanil-based anesthesia and those who received fentanyl-based anesthesia, the difference in the number of leucocytes may be mainly due to the kinetics of neutrophils.

One explanation for why the neutrophil count was higher in the fentanyl group is an increase in release of neutrophils from bone marrow by inflammatory cytokines in the fentanyl group. Terashima et al. [9] demonstrated that administration of IL-8 rapidly increased the numbers of granulocytes and polymorphonuclear leukocytes without affecting transit time through both the mitotic and post-mitotic pools of bone marrow. In addition to G-CSF, IL-6 and IL-3 stimulate granulopoiesis *in vivo* [10]. Remifentanil attenuates expression of pro-inflammatory cytokines including TNF-α, IL-6, and IL-8 *in vitro* [8]. Another *in vitro* study demonstrated decreased neutrophil transmigration through the endothelial cell monolayer by co-incubation with remifentanil [11], and this suggests that a smaller number of neutrophils migrated to damaged tissue from microvessels in the remifentanil group than in the fentanyl group. The possibility that a low number of neutrophils reached damaged tissue in the remifentanil group suggests that the amount of cytokines released from neutrophils recruited in the damaged site may have been lower in the remifentanil group than in the fentanyl group. Among patients who underwent cardiac surgery, patients who received fentanyl-based anesthesia had a higher level of stress hormone and higher levels of proinflammatory cytokines compared with patients who received remifentanil-based anesthesia [12]. In the present study, the numbers of leucocytes and neutrophils were both correlated with the duration of surgery among the patients in the remifentanil group. Long duration of surgery may be related to surgical stress. In the present study, one possible reason for the difference in the number of neutrophils between the remifentanil group and fentanyl group is a difference in production of neutrophils in bone marrow introduced by differences in surgical stress and inflammatory cytokines.

There have only been a few studies on the relationship between remifentanil and immunosuppression. Studies in vitro demonstrated that high-dose remifentanil suppresses the release of cytokines from neutrophils and monocytes that had been stimulated by LPS [8]. Very low-dose remifentanil did not suppress NK-cell activity in healthy human volunteers [13]. A study in rats demonstrated that continuous infusion of remifentanil inhibited the proliferation of splenocytes induced by concanavalin A [14]. Although the reductions in the elevation of leucocytes and neutrophils in the remifentanil group may have been related to reduction in the proliferation of neutrophils, whether suppression of increases in the numbers of leucocytes and neutrophils directly causes immunosuppression is unknown. General anesthetic agents induce different inflammatory responses [6, 7]. Sevoflurane has a smaller effect on the neutrophil count than desflurane [7]. The Mac-hour of sevoflurane in the remifentanil group was significantly lower than that in the fentanyl group, although the difference was very small. We believe that the effect of sevoflurane on the results may have been small.

In conclusion, remifentanil-based anesthesia attenuated postoperative leucocyte and neutrophil counts. It is unknown whether this phenomenon indicates the possibility of immunosuppression. Further studies are required.

## Consent

Written informed consent was obtained from the patient for publication of case report and any accompanying images. A copy of written consent is available for review by the editor-in-chief of this journal.
